# A New biological proxy for deep-sea paleo-oxygen: Pores of epifaunal benthic foraminifera

**DOI:** 10.1038/s41598-018-27793-4

**Published:** 2018-06-21

**Authors:** Anthony E. Rathburn, Jake Willingham, Wiebke Ziebis, Ashley M. Burkett, Bruce H. Corliss

**Affiliations:** 1 Earth and Environmental Systems, Indiana State University, Terre Haute, IN 47808 USA; 2 Integrative Oceanography Division, Scripps Institution of Oceanography, 9500 Gilman Drive, La Jolla, CA 92093-0218 USA; 3 Department of Biological Sciences, Marine Environmental Biology, University of Southern California, Los Angeles, CA 90089 USA; 4 Graduate School of Oceanography, University of Rhode Island, Narragansett, RI 02882 USA; 5 Present Address: Boone Pickens School of Geology, Oklahoma State University, 105 Noble Research Center, Stillwater, OK 74075 USA; 6 Present Address: Department of Geological Sciences, California State University, Bakersfield, CA 93311 USA

## Abstract

The negative consequences of fossil fuel burning for the oceans will likely include warming, acidification and deoxygenation, yet predicting future deoxygenation is difficult. Sensitive proxies for oxygen concentrations in ancient deep-ocean bottom-waters are needed to learn from patterns of marine deoxygenation during global warming conditions in the geological past. Understanding of past oxygenation effects related to climate change will better inform us about future patterns of deoxygenation. Here we describe a new, quantitative biological proxy for determining ocean paleo-oxygen concentrations: the surface area of pores (used for gas exchange) in the tests of deep-sea benthic foraminifera collected alive from 22 locations (water depths: 400 to 4100 m) at oxygen levels ranging from ~ 2 to ~ 277 μmol/l. This new proxy is based on species that are widely distributed geographically, bathymetrically and chronologically, and therefore should have broad applications. Our calibration demonstrates a strong, negative logarithmic correlation between bottom-water oxygen concentrations and pore surface area, indicating that pore surface area of fossil epifaunal benthic foraminifera can be used to reconstruct past changes in deep ocean oxygen and redox levels.

## Introduction

**Figure 1:**
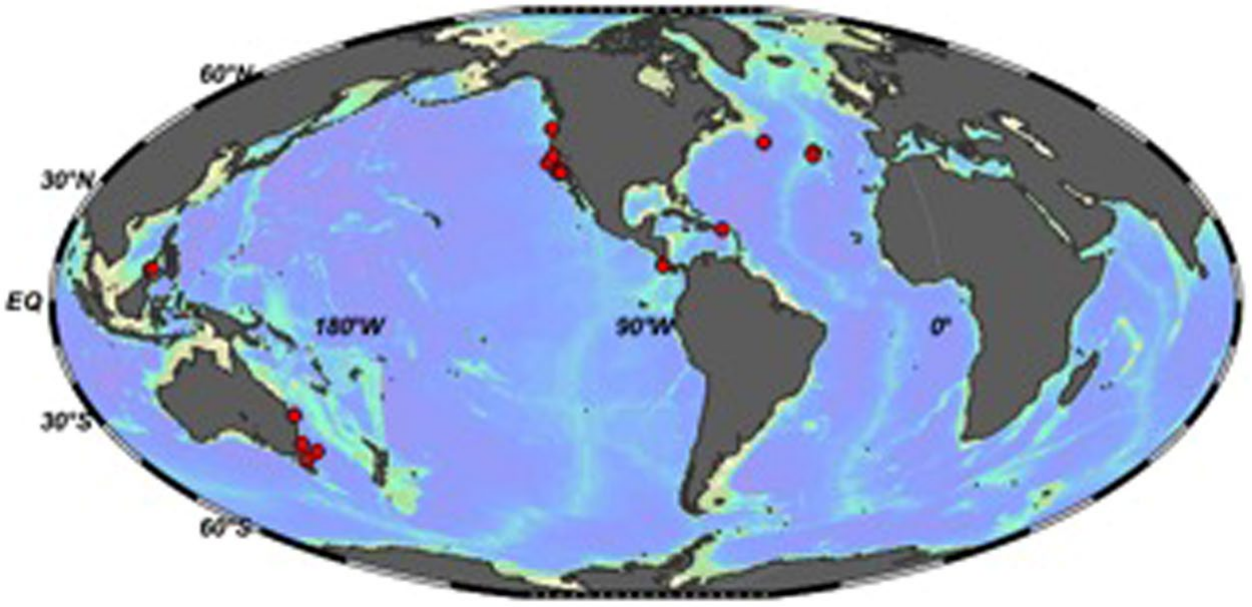
Global map showing site locations. Red dots represent locations where living/recently living individuals of deep-sea benthic foraminifera were collected for this study. The map was produced using Ocean Data View (Schlitzer, R., Ocean Data View, odv.awi.de, 2017).

Oxygen in intermediate and deep ocean waters is primarily taken up at high latitudes, where the surface ocean equilibrates with the atmosphere. Oxygenated waters are subsequently transported away from their source region via deep ocean circulation^1^. Removal of dissolved oxygen mainly occurs though oxidation of organic matter along oceanic circulation pathways, especially in regions of high biological productivity. This process ultimately determines dissolved oxygen concentrations below the thermocline, creating pronounced geographic and bathymetric variability in deep ocean oxygen concentrations, ranging from anoxic to fully oxic (100% saturated)^1,2^. Average dissolved oxygen in the deep ocean has also changed through geologic time, with such changes in oceanic dissolved oxygen inventories linked to total respired carbon (i.e., the “biological pump”), and in turn, to atmospheric CO_2_ concentrations^2,3^. By examining the effects of global warming in the geological past, we can learn about potential patterns of marine deoxygenation in the future. Quantifying marine dissolved oxygen content in ancient deep oceans using currently available proxies is typically problematic because of the complex interplay between physio-chemical (temperature, ocean circulation patterns, oxygen solubility, continental weathering and runoff) and biological parameters (primary and export productivity, depth and intensity of organic remineralization) and their equally complex influences on oxygen concentrations, biogeochemical cycles and seafloor population dynamics^2,4,5^. In this study, using specimens from a wide variety of locations, water depths and oxygenation conditions (Table 1; Fig. 1), we investigate the hypothesis that epifaunal (living at or above the sediment-water interface) deep-sea benthic foraminiferal test morphology (surface area of pores on their calcareous tests) responds to bottom water oxygen availability.

**Table 1:** Site location information.

Region	Oxygen (μmol/l)	# Specimens	Depth (m)
Hydrate Ridge, Pacific Northwest	10.72	9	775
Southern California Bight	27.14	8	1005
Southern California Bight	25.10	9	757
Southern California Bight	26.16	4	910
Southern California Bight	32.83	1	1510
Southern California Bight	25.04	2	665
Southern California Bight	25.34	2	815
Costa Rican Margin	1.79	3	400
Costa Rican Margin	32.15	8	997
Californian Margin	41.09	1	1050
Monterey Bay, California	44.65	2	1006
Sulu Sea, Philippines	78.55	1	510
Sulu Sea, Philippines	55.96	3	1995
Puerto Rican Margin	89.32	12	2007
Station M, Californian Margin	142.91	10	4100
Tasman Sea, Australia	202.75	4	580
Tasman Sea, Australia	201.85	1	747
Tasman Sea, Australia	201.41	2	770
Tasman Sea, Australia	170.59	2	1250
North Atlantic Margin	275.44	4	3682
North Atlantic Margin	267.95	4	2033
North Atlantic Margin	268.39	4	2130

General location descriptions where specimens were collected for this study, including the number of specimens examined from each site, bottom water oxygen concentrations and water depths.

Production of information on past ocean oxygenation has been slow, due at least in part to the lack of sensitive redox proxies^2,4^. Geochemical proxies of marine oxygenation provide information about oxygen conditions in pore waters within sediments or on global ocean oxygenation, and most can not yield insights about local/regional bottom water oxygenation. Relatively new redox proxies, including I/Ca^6–8^ and δ^13^C^9^ have been applied mainly to planktonic foraminifera or infaunal benthic foraminifera, which may not reflect bottom water conditions. Multiple species analyses using I/Ca and/or δ^13^C show promise, but may be complicated by subsurface pore water chemistry^10^. Well established redox proxies based on concentrations of S, Fe, Mo, and U in bulk sediment can trace the more extreme changes in oxygen concentration in remote Earth history, such as during the Archean^11,12^. However, these geochemical proxies may not be sufficiently sensitive to assess oxygenation variations under globally well-oxygenated conditions, including those of the Cenozoic^2,13^. Diagenetic alteration is another challenge, compromising the preservation of original geochemical signals when the carrier of the proxy is exposed to different redox conditions during burial^14^. Oxygenation proxies using microfossil assemblages or indicator species typically include abundances of infaunal taxa that reside within sediments, and may not reflect bottom water conditions^4^. In addition, species abundances and seafloor ecosystems typically have similar responses to multiple variables (including organic matter; i.e., TROX model^4^), making it difficult to isolate influences of changes in paleo-oxygenation^4,15^. Here, we describe a new, biological proxy, the use of which may lead to increased understanding of deoxygenation during global warming, its geographic and bathymetric variations, and its effects on marine biota, by analyzing oxygen concentrations during global warming episodes of the past.

Global warming is predicted to impact marine ecosystems in complex ways, including through a reduction in oxygen dissolved in ocean waters^2,16,17^. A reduction in available oxygen will have severe consequences for life in the oceans because eukaryotes respire oxygen, and oxygen is involved in the cycling of biologically important elements, including carbon and nitrogen^13,17^. Higher ocean temperatures lead to a reduction in oxygen solubility which results in decreases in dissolved oxygen in marine waters^17,18^. Some models predict that a warmer global climate will enhance nutrient runoff into the oceans^19^, leading to increases in productivity and greater organic matter delivery to the seafloor. Decomposition of organic matter consumes oxygen, and as a result, enhanced productivity can further reduce dissolved oxygen concentrations in bottom waters^2,3,14^. Increased biological metabolic rates caused by increases in water temperature may also result in enhanced remineralization of organic matter^20^. Oxygen Minimum Zones (OMZs) are predicted to increase in size in many areas of a warmer globe^17^, and predicted stratification of ocean waters under warmer conditions may also lead to reductions in productivity, gas exchange with surface waters and deep-ocean ventilation^17,21^. Elsewhere, warmer Earth changes in ocean circulation and upwelling may change productivity and organic matter export to the seafloor^22^, altering oxygen availability in other ways. Consequently, forecasting the extent, rate and intensity of potential future ocean deoxygenation remains challenging^2^. The biological proxy described herein represents the critical first step in establishing this morphological technique as a means to assess changes in bottom water oxygenation of the past.

### Benthic Foraminifera and Their Pores

Benthic foraminifera are protists which form tests that may be either proteinaceous, constructed of agglutinated particles, or made from secreted calcium carbonate. These organisms have a widespread distribution throughout the oceans and calcareous and agglutinated specimens are common in the fossil record^4^. Benthic foraminifera have microhabitat preferences, with epifaunal taxa living at the sediment-water interface or attached to hard substrates such as rocks, pebbles or spicules, whereas infaunal taxa live within the upper 10 cm of deep ocean sediments^23–25^. Where there is high input of organic matter, dissolved oxygen in interstitial water decreases rapidly within the upper few mm of sediment, whereas areas with a lower organic carbon flux to the seafloor generally have greater penetration depths of interstitial oxygen within sediments^23,25,26^. Calcareous epifaunal taxa, especially those living attached to objects above the sediment water interface^27,28^, are the focus of this study because they are in direct contact with overlying bottom waters, are widely distributed^29^, and are not influenced by oxygen gradients within seafloor sediments^15,23,25^. Such epifaunal foraminifera, e.g., *Cibicidoides wuellerstorfi*, can be abundant in oxygen deficient habitats^4,28^ as well as in well-oxygenated environments^4,24,27^.

**Figure 2:**
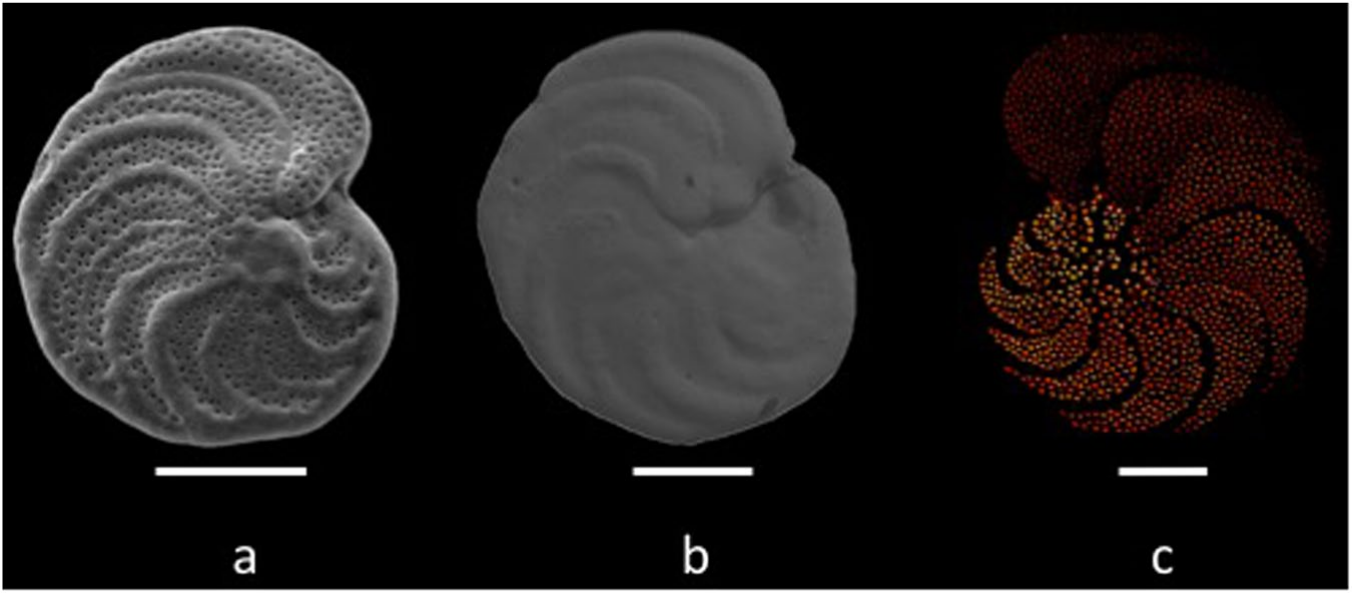
Images of 3 epifaunal foraminiferal specimens. Image A: SEM photo of *Cibicidoides wuellerstorfi* collected alive from a site with dissolved bottom water oxygen concentration (BWDO) of 27.7 μmol/L, Southern California Margin. Image B: SEM photo of *C*. *wuellerstorfi* collected alive at a BWDO of 200.1 μmol/L, Southeastern Australian Margin. Note the pronounced difference in pore abundance between specimens A and B. Image C: pores highlighted from an SEM image of *C*. *wuellerstorfi* (using Adobe Photoshop ©) collected alive at BWDO of 27.2 μmol/L, Southern California Margin. Scale bars are 200 micrometers.

Many calcareous foraminiferal taxa have pores over the entire test or portions of it (Fig. 2). Although our understanding of the function of these pores is incomplete^30^, field and laboratory observations suggest that surface pores on the test are used for gas exchange^31–33^, with size and number of pores on benthic foraminifera from oxygen-poor environments tending to be higher than those of specimens from well-oxygenated habitats^34–38^. In laboratory studies, chambers of foraminifera under oxygen-poor conditions have larger pores (and greater pore surface area) than chambers grown at greater oxygen availability^38^. The number of pores on the tests of at least some infaunal taxa is inversely related to ambient bottom water oxygen concentrations^35,36^, but the size of pore openings (and therefore functional surface area for gas exchange) can be variable^38^. The functionality of pores distributed over tests of mobile infaunal taxa may also be variable^30^. The epifaunal species *Cibicides lobatulus*, has large ventral pores (ventral is the side of test attachment), which it uses for adhesion to hard substrates^39^. Measurements of pore surface area in our study account for variations in both abundance and size of pores, and restriction of analyses to the dorsal side (the side exposed to bottom water, not used for attachment) of epifaunal tests omits pores used for adhesion and limits analyses to only those pores exposed to bottom water (not pore water within sediments).

## Results

### Pore Surface Area and Ambient Oxygen

**Figure 3:**
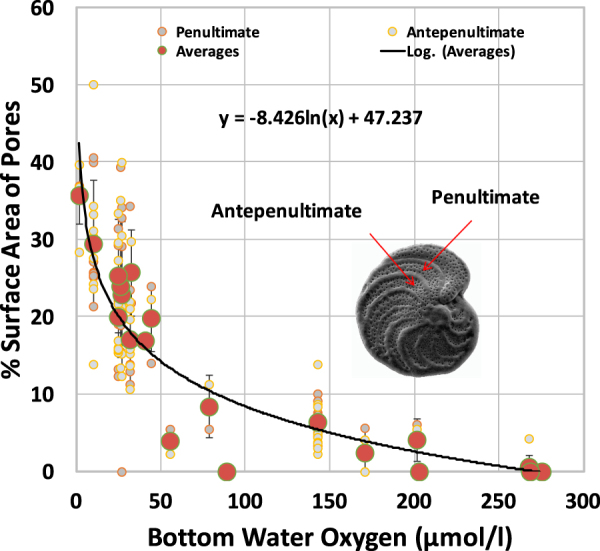
Relation between pore surface area on the penultimate and antepenultimate chambers of eipfaunal foraminifera and dissolved oxygen concentration in of ambient bottom waters. Large dots represent average values of specimens collected alive at each location; smaller dots represent individual values of specimen chambers. Lines with bars represent standard deviations.

Living/recently living specimens of the same morphological species, *C*. *wuellerstorfi*^29^, along with other epifaunal taxa, were analyzed from both well-oxygenated habitats and oxygen-poor environments (1.8 to 276.9 μmol/l; water depths from 400 to 4100 m; Table 1**)**. Within a single species the surface area of pores can range from zero to over 40% (Figs 2 and 3). The highest pore surface areas on the dorsal side (exposed to bottom water) occur in specimens from the lowest dissolved oxygen values in bottom waters (Fig. 3), independent of test size, location and water depth. Analysis of the combined average percentage of pore surface areas of penultimate and antepenultimate chambers of 96 epifaunal tests (Fig. 3) yielded a strong, negative, logarithmic relationship with ambient bottom water oxygen such that:

1 $${\rm{Dissolved}}\,{\rm{oxygen}}\,{\rm{in}}\,{\rm{bottom}}\,{\rm{water}}={{\rm{e}}}^{(\mathrm{Pore} \% -47.237/-8.426)}$$

(Fig. 3; Supplementary Table 1).

We chose not to analyze the entire test or an entire chamber because of image distortions on the rounded edges of specimens (see methods section) and because of variability in pore distribution on chambers. In specimens from habitats with bottom water oxygen concentration values of 50 to 200 μmol/l, early chambers commonly are without pores, apparently covered by biologically produced, secondary calcite. Pore surface area in the last few larger chambers is obviously sufficient for respiration needs.

## Discussion

A negative correlation between pore surface area and ambient dissolved oxygen concentrations was expected based on previous observations of pore abundances related to oxygen levels^4^. Pores on the dorsal surface of epifaunal foraminiferal tests most probably are used for gas acquisition^31–33^, so the logarithmic relationship between epifaunal pore surface area and ambient oxygen availability indicates that a greater test pore surface area is needed for respiration as dissolved oxygen in the bottom water decreases. We speculate that this logarithmic relationship results from changes in metabolic requirements as ambient oxygen availability decreases. It has been previously noted that the average size of foraminiferal specimens in assemblages living in oxygen-poor environments is smaller, potentially because of oxygen availability (due to surface/volume ratios)^4,25^, or because they reach reproductive maturity faster (because of the typical abundance of food in oxygen-poor habitats)^15^. In our data set, there was no relationship between specimen size and oxygen availability. We suggest that because these epifaunal foraminifera are able to adjust the size and number of pores, there is less of a need for these species to adjust their size and volume. At least some epifaunal foraminifera, including *C*. *wuellerstorfi*, are able to obtain sufficient oxygen through test pores to live in environments with dissolved oxygen values as low as 1.8 μmol/l (Fig. 3; Table 1), but there are few, if any pores on epifaunal tests collected from habitats with bottom water oxygen levels above about 200–250 μmol/l. At dissolved oxygen values above this threshold, epifaunal foraminifera do not require pores on the dorsal side (side that is exposed to bottom water; side that is not attached) of their test and must obtain adequate oxygen through the aperture (the primary opening in the test).

For the entire data set, the average site pore surface area percentage only deviates from the logarithmic line by an average of about 0.5 (Fig. 3). Below 50 μmol/l, average site pore surface area percentages deviate from the logarithmic line by an average of 2.7, predicting bottom water oxygen values within about 5 to 12 μmol/l. This represents higher resolution than most existing proxies in oxygen-poor conditions^4^. Inter-specimen variability in pore surface area may result from differences in the metabolic requirements between individuals and/or differences in microenvironments surrounding individuals. In general, pore surface areas of penultimate and antepenultimate chambers of the same individual are similar (Fig. 3; Supplementary Table 1). Any differences may result from temporal changes in ambient conditions between construction of the two chambers, secondary calcite precipitated over some pores, or metabolic changes requiring a difference in access to oxygen. Given that significant deep-sea oxygenation changes are unlikely on the time scales that foraminifera generate two successive chambers, we speculate that any appreciable differences in pore surface area between the penultimate and antepenultimate chambers result from secondary calcite and/or metabolic changes.

Principal Components Analysis eigenvectors in a biplot comparing available environmental variable data (total organic carbon (TOC), bottom water temperature and water depth) showed that dissolved oxygen had the strongest relationship with pore surface area (Supplementary Fig. 1; Supplementary Tables 2–4). The inverse correlation between TOC and dissolved oxygen in most natural settings is also reflected in the analyses. There is no evidence we are aware of to suggest that TOC has any influence on test pore surface area. Three pore surface area groups (suboxic (≤22 μmol/l), dysoxic ( > 22 μmol/l and < 89 μmol/l), and oxic (≥89 μmol/l)) were statistically significant from each other (Methods; Supplementary Table 5). Nitrate data were not available for most sites.

Nitrate availability in bottom waters has been correlated to pore abundances in at least some infaunal benthic foraminifera^40^. Nitrate respiration occurs in many infaunal benthic foraminiferal taxa^41–44^, and a correlation between ambient nitrate availability and the number of pores was reported for the infaunal species, *Bolivina spissa*^45^. However, the number of pores on tests of the infaunal species, *Bolivina pacifica*, *Fursenkoina mexicana* and *Globobulimina turgida*, is more closely related to oxygen availability than to nitrate concentration^35,36^. Unlike many infaunal taxa, epifaunal foraminifera do not live or migrate through anoxic pore waters and are not likely to have evolved the mechanisms required to respire/store nitrogen^41^. For example, *Cibicidoides pachyderma*, an epifaunal species similar in appearance and closely related to *Cibicidoides wuellerstorfi*^46^, does not contain stored nitrate, indicating that this species does not obtain oxygen from nitrate^41^. To our knowledge, there is no evidence showing that deep-sea epifaunal species of *Cibicidoides*, *Planulina* or *Cibicides* use nitrate as a means of respiration.

Taxa that were used for analyses in this study have been wide spread in marine environments over a broad range of water depths since the mid-Miocene. Related taxa and recognizable epifaunal morphologies extend back to the late Cretaceous^4,47^. Provided that a sufficient section of the penultimate and/or antepenultimate chamber of fossil epifaunal foraminiferal specimens is preserved, and any authigenic overgrowths removed, we propose that this new proxy can be used to assess the history of paleo-oxygenation. It would be important to choose epifaunal taxa/morphologies, and if possible, analysis of multiple specimens per interval. Analyses of epifaunal specimens can provide information about bottom water oxygenation, avoiding the influence of pore water or movement of infaunal taxa within a subsurface oxygen gradient.

We argue that our analyses of living, epifaunal, deep-sea benthic foraminifera revealed ecophenotypic responses to oxygen availability, providing a robust mechanism for modern and paleo-oxygenation evaluations. The direct relationship between the surface area of pores on epifaunal, deep-sea foraminiferal tests and ambient bottom water oxygen thus provides a new, quantitative means to assess bottom water oxygenation of Cenozoic and Late Cretaceous oceans.

## Methods

The epifaunal taxa, *Cibicidoides wuellerstorfi*, *Cibicides lobatulus*, and *Planulina* sp., were chosen for this study because of their cosmopolitan distribution and genetic homogeneity in the deep-sea over a wide range of oxygen values^28,29,46^. These species have plano-convex or lenticular shaped tests typical of epifaunal taxa^15,23,47^. Benthic foraminiferal specimens (96) were collected from 22 locations (water depths from 400 to 4100 m) with oxygen levels ranging from 1.8 to 276.9 μmol/l (Table 1. Figure 1). Specimens were manually removed (on board the ship) from living attachment on rocks, worm tubes or artificial substrates, and then frozen at −80 °C or picked from samples that had been preserved in 4% buffered formaldehyde solution and stained with Rose Bengal (to distinguish living and recently living individuals). Rose Bengal stains foraminiferal protoplasm red, indicative of the presence of protein-rich material inside the test. The limitations of Rose Bengal staining are well known, and using conservative methods, this commonly used stain provides indication of living or recently living individuals^48^. Restriction of the data set to living or recently living specimens avoids analysis of individuals that were not associated with ambient conditions in time and space.

Scanning Electron Microscope (SEM) images were taken of the dorsal side of each specimen (which is the side exposed to bottom water), and percentages of pore areas of the test and penultimate and antepenultimate chambers were determined. Comparisons of dissolved oxygen values with surface pore percentages of penultimate and antepenultimate chambers showed that both chambers separately and combined had similar relationships to ambient oxygen concentration. The penultimate chamber of a foraminiferal test is the next-to-the-last chamber and the antepenultimate chamber is the chamber formed immediately prior to the penultimate chamber (Fig. 3). Since the last (ultimate) chamber of specimens is often damaged, especially in fossil specimens, use of the penultimate and antepenultimate chambers in fossil specimens provides a more reliable and relevant method to obtain pore percentage data. The primary difficulty in analyzing the surface area of pores for an entire chamber or chambers, or the entire dorsal side of the test is the three-dimensional nature of foraminiferal tests; SEM images of the pores occurring along the curved edges around the periphery of even relatively thin planispiral tests tend to distort the surface area of the pore opening. As a result, analysis of a uniform surface area (100 × 50 µm (5,000 µm^2^) that fit chambers of all specimens and located in the middle, flat portion of the chamber yields a more representative surface area percentage than including the curved portion of the test. In addition, pores of early chambers may be covered with secondary, biologically produced, calcite or obscured by subsequent chambers, reducing the overall average and potentially misrepresenting pore surface area relationships with existing conditions. In their development of methodology to analyze the pores of a shallow-water genus, Petersen *et al*. concluded that it is best to focus on subset windows of chambers of the same ontogenetic stage for comparisons^49^.

The surface area percentage of pores and the number of pores on the penultimate, antepenultimate chambers, and the entire dorsal side of the test were determined using SEM images, Adobe Photoshop to isolate pores (Fig. 2), and ArcGIS software (version 10.1, advanced) to quantify pores pixels using the *Iso Cluster Unsupervised Classification* tool in ArcMap. These software programs were chosen because of their accuracy, ease of use, and general availability. Surface area percentages were determined by analyzing the pore surface area within a standard sized square (chamber box) on both the penultimate and antepenultimate chambers. Pore percentages of each chamber were averaged for each specimen. The best fit equation (Equation (1)) was based upon average site values. Bottom water oxygen values were determined measuring high-resolution oxygen profiles from the overlying water into the sediment in multicorer tubes taken at the collection site using amperometric oxygen microelectrodes^50^, or an oxygen sensor mounted on a CTD or submersible. Data have been included as supplementary information in Supplementary Table 1.

A non-parametric analysis of variance was performed on all 96 specimens. Based on the dissolved oxygen concentration from which each specimen was collected, epifaunal individuals were separated into one of three oxygenic subgroups (suboxic (≤22 μmol/l), dysoxic (>22 μmol/l and <89 μmol/l), and oxic (≥89 μmol/l)). Kruskal-Wallis one-way analysis of variance was used to compare all three groups to each other, while Mann-Whitney analysis provided a means to compare individual groups to each other. These analyses determined that each group of specimens was statistically (p < 0.005) independent (not-influenced) by the other two groups (Supplementary Table 5). Kruskal-Wallis and Mann-Whitney analyses were accomplished using IBM SPSS and verified on R.

Data were also statistically analyzed using a subset of data. Since TOC data were not available for all sites (and not relevant for 28% of the sites where live specimens were removed from substrates elevated well above the sediment-water interface). As a result, the first subset of data included 26 locations for which TOC, bottom-water temperature and oxygen, and average pore percentages were available. This 26-point data set was then subjected to principle component analysis in R (Supplementary Fig. 1; Supplementary Table 4). Illustrated by the PCA biplot, eigenvectors assigned to average pore surface area percentage and dissolved oxygen are diametric, meaning that they are inversely correlated. This same diametric trend can be observed between the TOC and dissolved oxygen eigenvectors, which is to be expected given that oxygen is consumed in the metabolization of organic carbon. Calculation of Pearson Correlation Coefficients to determine linear relationships between the variables also confirmed that pore surface area and dissolved oxygen had the strongest relationship of the variables examined (Supplementary Tables 2 and 3). Data generated or analyzed during this study have been included in the supplementary information files. Additional information is available from the corresponding author upon reasonable request.

## Electronic supplementary material

Supplementary Data Figure 1 and Tables 1–5
