# Supplementary material for: A New biological proxy for deep-sea paleo-oxygen: Pores of epifaunal benthic foraminifera
Source: Sci Rep. 2018 Jun 21;8:9456. doi: 10.1038/s41598-018-27793-4 (PMC6013501; doi:10.1038/s41598-018-27793-4)
Supplement: Supplementary file 1 — Supplementary Data Figure 1 and Tables 1–5 [file 41598_2018_27793_MOESM1_ESM.docx]

**A New biological proxy for deep-sea paleo-oxygen: Pores of epifaunal benthic foraminifera**

**Anthony E. Rathburn^1,2*^, Jake Willingham^1^, Wiebke Ziebis^3^, Ashley Burkett^1**^, and Bruce H. Corliss^4^,**

*^1^ Earth and Environmental Systems, Indiana State University, Terre Haute, IN 47808, USA*

*^2^ Integrative Oceanography Division, Scripps Institution of Oceanography, 9500 Gilman Drive La Jolla, CA 92093-0218, USA*

*^3^Department of Biological Sciences, Marine Environmental Biology, University of Southern California, Los Angeles, CA 90089, USA.*

*^4^Graduate School of Oceanography, University of Rhode Island, Narragansett, RI 02882 USA*

*^*^ Corresponding Author currently at Department of Geological Sciences, California State University Bakersfield, CA 93311 USA arathburn@csub.edu*

*** Currently at Boone Pickens School of Geology, Oklahoma State University, 105 Noble Research Center, Stillwater, OK 7407*

**Supplementary Data Tables and Figures.**

**Supplementary Figure 1.** Statistical analyses were run on two subsets of the data. The first subset of data included 26 data points for which TOC, bottom-water temperature and oxygen, and average pore percentages were available. This 26-point data set was then subjected to principle component analysis in R generating Supplementary Figure 1. Illustrated by the PCA biplot, eigenvectors assigned to average pore percentage and dissolved oxygen are diametric, meaning that they are inversely correlated. This same diametric trend can be observed between the TOC and dissolved oxygen eigenvectors, which is to be expected given the usage of oxygen in metabolizing of organic carbon. The inverse correlation between pore percentage and oxygen was further confirmed through the calculation of a Pearson Correlation Coefficient to determine the linear relationship between the variables. Results can be found below and confirm the findings of the PCA analysis.


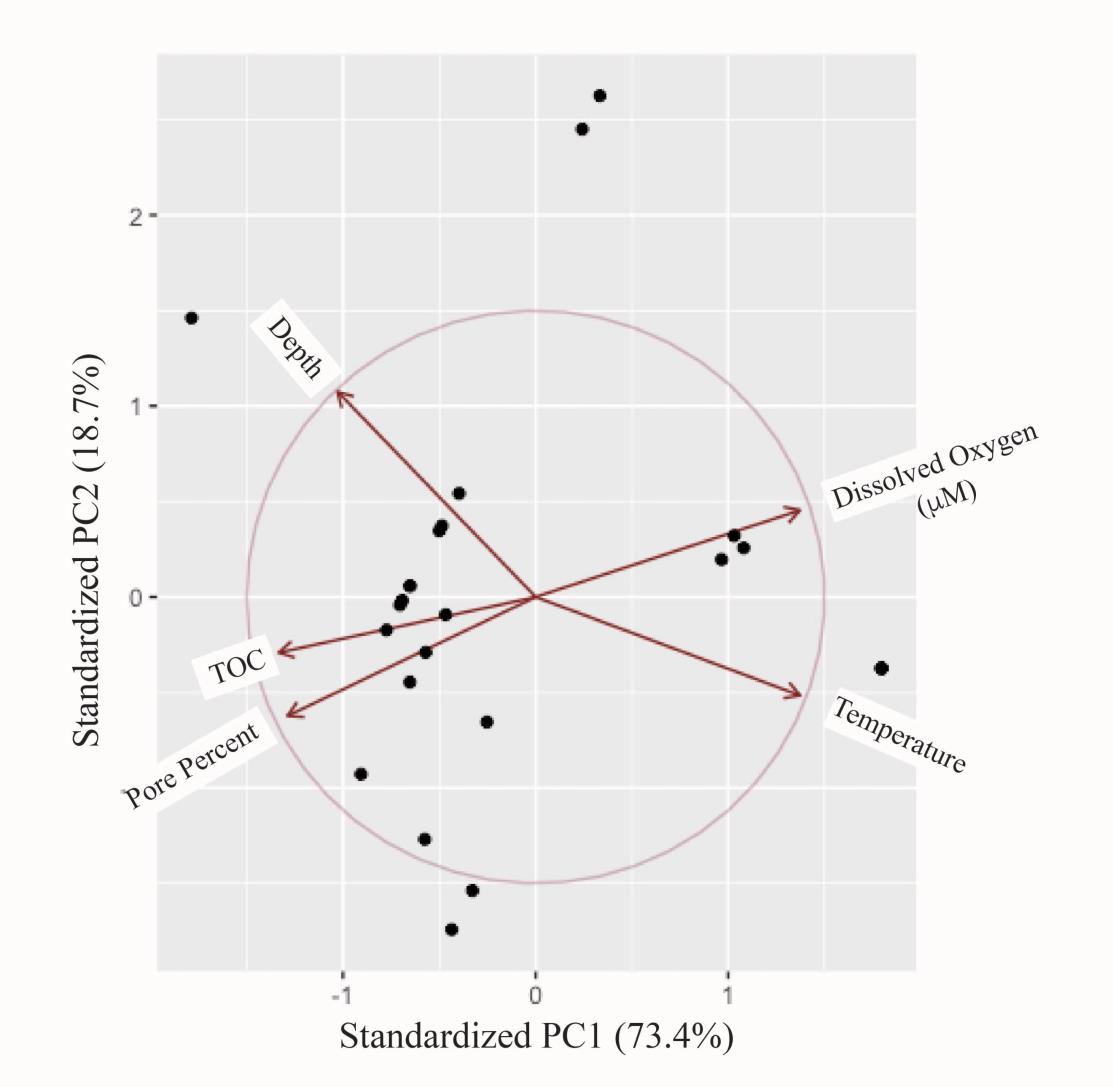


**
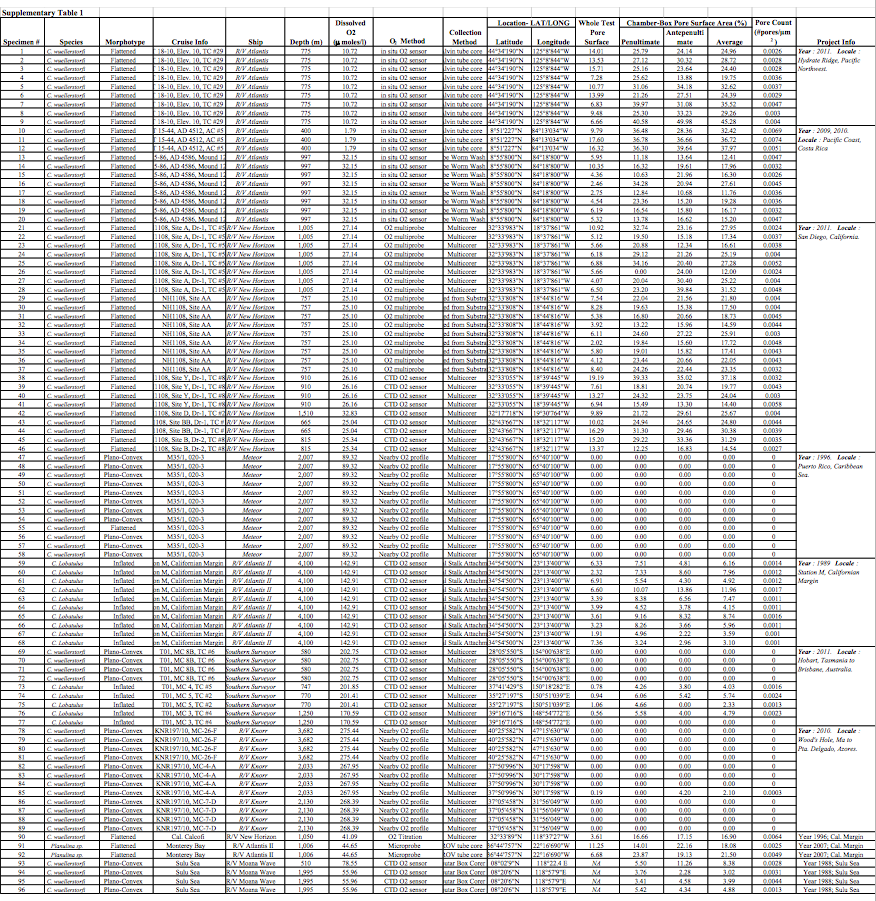
**

**Table 1.** Data Table showing Species analyzed, Morphotype, Cruise, Water depth, Dissolved Bottom water Oxygen, Collection method, Latitude and Longitude, Whole test pore surface area percentages, Penultimate chamber pore surface area percentages, Antepenultimate chamber pore surface area percentages, Average of Penultimate and Antepenultimate pore surface area percentages, Pore count, and Sample locality.

**Supplementary Table 2**

The results of the Pearson correlation coefficients are strictly for a linear relationship. As has been shown in Figure 3, a logarithmic trend line seems to be a better fit for the average pore percentage and oxygen data. Therefore, each variable was plotted against each other, a logarithmic trend line was added and an R2 value determined for each line.

|   **Variable 1** |    **Variable 2** | **Pearson Correlation Coefficient** |    **n=** |
| --- | --- | --- | --- |
|   Average Pore Percentage |    Oxygen | -0.88 |    26 |
|   Average Pore Percentage |    TOC | 0.77 |    26 |
|   TOC |    Oxygen | -0.82 |    26 |
|   Average Pore Percentage   |    Oxygen    | -0.75 |    97    |
|   **Variable 1**   |    **Variable 2**    | **R2** |    **n=**    |
| Average Pore Percentage | Oxygen | 0.77 | 26 |
|   Average Pore Percentage |    TOC | 0.63 |    26 |
|   TOC   |    Oxygen    | 0.65 |    26    |
| Average Pore Percentage   | Oxygen    | 0.75 | 97    |

**Supplementary Table 3**

There is a measurable relationship between dissolved oxygen and average pore percentage of benthic foraminiferal calcite. A PCA analysis indicates this relationship and it is further confirmed through calculations of Pearson’s correlation coefficients (linear) and R2 values of logarithmic trend lines. The strongest Pearson correlation coefficients and R2 values belong to comparisons of pore parentage and oxygen comparisons. Although there is an R2 of 0.63 between pore percentage and TOC it may be argued that any apparent relationship between TOC and pore percentage is a byproduct of the inverse relationship of TOC and dissolved oxygen.

**Supplementary Table 4a**

Numeric results from the PCA analysis:

|  |    **PC1** | 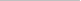  **PC2** | **PC3** |  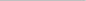  **PC4** |   **PC5** |
| --- | --- | --- | --- | --- | --- |
|   **Depth**   |    -0.3576307    | 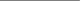  0.7416385  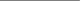 | -0.07036532 |  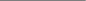  0.25888394   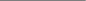 |   0.500100467   |
| **TOC** | -0.4638053 | -0.2008326 | -0.85042393 | -0.01612205 | -0.145155629 |
|   **Temperature**   |    0.4777707    | 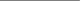  -0.3559989  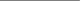 | -0.30164445 |  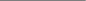  0.23106701   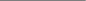 |   0.707544043   |
| **Dissolved O2**   | 0.4774501    | 0.3132905 | -0.26446146 | 0.61298610 | -0.477701412 |
| **Pore Average** | -0.4480642 | -0.4298284 | 0.33301444 | 0.70963037 | -0.003486253 |

**Supplementary Table 4b**

Importance of components %s:

|  | **PC1** | **PC2** | **PC3** | **PC4** | **PC5** |
| --- | --- | --- | --- | --- | --- |
| Standard deviation | 1.9152 | 0.9659 | 0.48852 | 0.35862 | 0.17813 |
| Proportion of Variance | 0.7336 | 0.1866 | 0.04773 | 0.02572 | 0.00635 |
| Cumulative Proportion | 0.7336 | 0.9202 | 0.96793 | 0.99365 | 1.00000 |

**Supplementary Table 5.**

*Table showing number of specimens in each oxygen group (n), the normality of the three oxygen groups, and the results of the statistical analyses performed. Both the Kruskal- Wallis and Mann-Whitney Tests of variance showed that all three groups (suboxic, dysoxic, and oxic) were statistically significant from each other.*

|  | **# of Specimens (n)** | **Shapiro- Wilk Test of Normality** | **Kruskal- Wallis ANOVA** | **Mann-Whitney U Test** | | |
| --- | --- | --- | --- | --- | --- | --- |
|  |  |  |  | *Suboxic* | *Dysoxic* | *Oxic* |
| **Suboxic** | 12* | 0.93 | 2.20E-16 |  | 3.91E-05 | 1.43E-08 |
| **Dysoxic** | 41 | 0.77 |  | 3.91E-05 |  | 7.46E-15 |
| **Oxic** | 43 | 1.71E-08 |  | 1.43E-08 | 7.46E-15 |  |
|  | | | | | | |
| *An n >30 is needed to strengthen the significance of this group | | | | | | |
